# Temporal and spatial variation of potassium balance in agricultural land at national and regional levels in China

**DOI:** 10.1371/journal.pone.0184156

**Published:** 2017-09-05

**Authors:** Yingxia Liu, Jinchuan Ma, Wencheng Ding, Wentian He, Qiuliang Lei, Qiang Gao, Ping He

**Affiliations:** 1 Institute of Agricultural Resources and Regional Planning, Chinese Academy of Agricultural Sciences (CAAS), Beijing, China; 2 College of Resources and Environment, Jilin Agricultural University, Changchun, China; 3 International Plant Nutrition Institute (IPNI) China Program, CAAS-IPNI Joint Lab for Plant Nutrition Innovation Research, Beijing, China; Huazhong Agriculture University, CHINA

## Abstract

Linking potassium (K) balance to soil fertility creates a valuable indicator for sustainability assessment in agricultural land-use systems. It is crucial for the efficient use of K resources, food security and resource sustainability to realize soil K balance status in China. Therefore, temporal and spatial changes of K balance for farmland in China from 1980 to 2015 were analyzed at national and regional levels using statistical data and related parameters. At the national scale, K input increased from 6.78 Mt K_2_O in 1980 to 23.44 Mt K_2_O in 2015 with an average annual increment of 0.48 Mt K_2_O, and output changed from 8.10 Mt in 1980 to 21.31 Mt in 2015 with an average annual increment of 0.38 Mt K_2_O as well. On average, K balance was -24.17, -5.92, 21.31 and 19.50 kg K_2_O ha^-1^ in 1980s, 1990s, 2000s and 2010s, respectively. Moreover, the average balance of six regions was considerably different which were -21.37, 1.25, 13.70, -22.79, 99.22 and 7.18 kg K_2_O ha^-1^ from 1980 to 2015. The potassium use efficiency (KUE) decreased with time which were 127.09, 104.35, 87.69 and 89.69% in 1980s, 1990s, 2000s and 2010s, respectively, and the decline of slope could also reflect the variation tendency of KUE. Great variation of K balance across different regions demonstrated that fertilizer application and management practices need to be adjusted to local conditions.

## Introduction

Low soil fertility is one of the main constraints of agricultural productivity, so enhancing soil fertility is the basis for guaranteeing food security [1, 2]. The recommended means of improving soil fertility is by regulating farmland nutrient cycles and balance through rational fertilization [3]. Balanced fertilizer application can not only save resources, but also improve economic benefits. Nutrient balances (also known as nutrient budgets) are calculated by the difference between nutrient inputs and outputs of a system with predefined spatiotemporal boundaries [4]. Thus, they are generally expressed as an amount of nutrient per unit of area and time (e.g., kg ha^-1^ yr^-1^). Negative nutrient balances indicate that a system is losing nutrients, however in some cases, nutrients apparently accumulate (and might lead to nutrient losses if strongly in excess). Nutrient balances have been used extensively for improving natural resource management and/or for recommendations over the last decades [5–9]; they are useful tools as indicators of potential land degradation and for optimizing nutrient use in agricultural systems to maintain sustainability of soil nutrient resources and agricultural production [10, 11]. Therefore, maintaining the balance between plant nutrient inputs and outputs in agroecosystems has become very important for sustainable development and soil quality [12, 13].

To provide enough food for the continuously growing population in China, large quantities of fertilizers, mainly nitrogen (N) and phosphorus (P) fertilizers, have been used in agriculture [3, 14]. However, potassium (K), an essential element for crops, has often been neglected [15]. Potassium deficiency is a global problem [16] and there have been huge changes in soil K all over the world, from Europe [17, 18] to Africa [19, 20] to Asia [21, 22], and North America [23]. China suffered a serious soil K deficiency from 1961 to 1997 [24]. Liu et al. [25] indicated that soil K had been a limiting factor for sustainable agricultural production especially in some parts of northeast and north-central China. In recent years, soil K deficiency has been relieved with the increase in fertilizer K application and straw return. Shen et al. [13] indicated that although the soil K balance was deficient in the period from 1993 to 2001, the situation was improved from 1999 to 2001. Several studies also showed that significant soil K surpluses were observed in vegetable production in Hebei [26], Shandong [27] and Jiangsu provinces [3].

Previous studies about K balance have mainly focused on specific experimental sites [15, 28–31] or short-term observations at national level [32], but it is difficult to show in-depth analysis of element balances based on specific experimental sites or one-year studies [3]. Although a negative K balance might not occur in the short term, long-term negative K balance is still a potential problem [33]. Because nutrient balances can vary considerably from year to year, short-term studies providing limited information might be misleading [24]; Sheldrick et al. [24] also emphasized the importance of using nutrient balances at the national level which would help to develop national fertilizer policies including decisions on investments in fertilizer factories and the exploitation of local resources and minerals to supply nutrients. Information on temporal variability of K balances in agroecosystems at the national level could be of great benefit for policymakers and farmers in finding strategies and measures for reasonable use of K resources to ensure food security [13], as described in previous studies [24, 34–36].

The objective of this study was to evaluate the temporal and spatial variation in K balances and K use efficiency in China from 1980 to 2015. The results will help to identify regions with poor soil fertility and assist in putting forward strategies by rational nutrient management to improve low-yielding field.

## Materials and methods

### Study area

Based on 31 provincial data sets, gross soil K balance was calculated in mainland China excluding Hong Kong and Macao. China is divided into six regions according to geographical locations and China’s administrative divisions, as shown in S1 Table [32, 37].

### Data sources

The data used in this paper, such as chemical fertilizer, livestock numbers, crop yield, population number living in the countryside, planting area and cultivated land area, were obtained from the China Agriculture Statistical Report [38].

In this study, data pre-processing primarily included original data verification. The former steps included filling in the missing data, data validation and derivative data construction. After that, a reasonable database was built, which included provincial K balance data from 1980 to 2015. For the sake of analysis, we compared the K balance across four decades: 1980s (1980–1989), 1990s (1990–1999), 2000s (2000–2009) and 2010s (2010–2015).

Because of the enormous amount of data in the original database, it was difficult to identify the flawed data. Therefore, a strict rule was applied for those indicators: the data from adjacent years could not vary by more than 15%. The missing data could be filled in either by referring to a statistical yearbook/published literature or by using the data from adjacent years. However, some indicators that did not exist in the original database needed to be acquired from other indicators. For example, the amount of straw is a required indicator, but it can only be calculated by multiplying grain crop production by a literature survey-based conversion factor (the ratio of grain to straw), which is distinguishing for different crops. The parameters needed in this model for K balance calculations in Chinese farmland were explained in Liu et al. [39]; spatial variation of parameters was estimated by region.

### Soil K balance model description

The soil K balance model included the following inputs: chemical fertilizer, organic manure, atmospheric dry and wet deposition, irrigation and crop seeds (K_2_O, similarly hereinafter) [39]. In detail, the organic fertilizer resources were categorized by human and animal manure, straw and cake manure. The following outputs were used: crops removal (including grain and straw uptake) and nutrient loss (leaching and runoff loss).

#### Chemical fertilizer inputs

The ratio for K_2_O in compound fertilizer was different in different regions and years, as shown in S1 Table [32, 37].

#### Organic manure inputs

The organic manure resources mainly include human and livestock manure, straw return and cake manure. The human and livestock manure including the parameters of livestock feeding period, daily excretion, K_2_O content and application rates to field were listed in Table 1 [39–41]. The quantity of crop straw was calculated through grain yields and the ratio of grain to straw for each type of crops and the K contents in straw were collected from published studies (Table 2) [42]. The rates of straw return to fields in the 2000s and 2010s were published in Li and Jin [32] with 15% in NE and NW, 60% in NC and 30% in the MLYR, SE and SW. In the 1980s and 1990s, the crop straw was removed from fields and returned back into the soil with burned ashes to different extents. The straw return rates in the 1980s were about 1/3 of those in the 2000s, (5% in NE and NW, 20% in NC and 10% in the MLYR, SE and SW). In the 1990s, the straw return rate was 2/3 of those in the 2000s, (10% in NE and NW, 40% in NC and 20% in the MLYR, SE and SW). The cake production rate of different crops and the K content in cake manure were collected from published data (S2 Table) [39].

**Table 1:** Livestock feeding period, daily excretion, K content and percentage for field application (based on fresh).

Livestock	Feeding phase	Herd population structure (%)	Feeding period (d)	Daily excretion/urine and K_2_O content				Rate to field (%)		
				Excrement (kg/d)	Urine (kg/d)	Excrement (%)	Urine (%)	1980s	1990s	2000s, 2010s
Human			365	0.31	1.59	0.370	0.190	55	45	33
Dairy	Adult cattle	52.9	365	37.5	18.8	0.150	1.111	55	45	30
	Young dairy cattle	37.1	365	21.4	10.7	0.150	1.111	55	45	30
	Holstein calf	10	180	5.8	2.9	0.150	1.111	55	45	30
Beef	Heifers for slaughter	72.9	365	18.1	9.1	0.253	1.060	55	45	30
	Fattening cattle	15.9	270	12.1	6.0	0.253	1.060	55	45	30
	Holstein calf	11.2	120	4.0	2.0	0.253	1.060	55	45	30
Horse			365	10	5.01	0.460	0.820	55	45	44
Donkey			365	10	5.01	0.640	0.280	55	45	44
Mule			365	10	5.01	0.280	0.340	55	45	44
Pig	Breeding pigs	11	365	5.5		0.294		55	45	65
	Fattening pigs	52	180	4		0.294		55	45	65
	Piglets	37	90	1.5		0.294		55	45	65
Sheep and Goat	Adult goats	70	365	2.6		0.641		55	45	33
	Lambs	30	180	1.4		0.641		55	45	33
Poultry		-	210	0.13		0.715		55	45	45

**Table 2:** The ratio of grain to straw and the K content in air-dry straw.

Crops	Straw/Grain			K_2_O in straw (%)
	1980s	1990s	2000s, 2010s	
Rice	1	0.99	0.91	2.053
Wheat	1.54	1.48	1.26	1.472
Winter wheat	1.54	1.48	1.26	1.229
Maize	1.26	1.13	1	1.276
Sorghum	1.8	1.8	1.8	1.735
Millet	1.4	1.4	1.4	1.962
Barley	1.6	1.6	1.6	1.527
Other cereals	1.6	1.6	1.6	1.348

#### Other inputs

In addition to chemical fertilizer and organic manure, atmospheric dry and wet deposition, irrigation and crop seeds were analyzed as sources of farmland nutrients (S3 Table).

#### Arable crop outputs

The amount of arable crop outputs was the principal nutrient output in the agricultural system which was calculated through economic yield and K requirement for unit of economic yield of different crops (Table 3) [32, 39]. The arable crop outputs were categorized into five kinds of crops, including cereal crops, fruit and vegetables (fruit, vegetable and melons), oil crops (oil crops and beans), industrial crops (sugar beet, sugar cane, fiber crops, cotton and tobacco) and root crops (sweet potato and potato).

**Table 3:** K requirement for unit of economic yield of different crops.

Crops	Content ^a^ (kg K_2_O t^-1^)	Crops	Content ^a^ (kg K_2_O t^-1^)
Wheat		Peanut	43.27
Winter wheat	27.14	Sunflower	93.22
Spring wheat	29.02	Rape	57.4
Maize		Sesame	59.8
Spring maize	18.44	Other oil crops	72.1
Summer maize	19.16	Vegetables	4.85
Rice		Fruit	6
Early season rice	21.01	Melons	6.5
Middle-season rice	22.11	Flue-cured tobacco	75.65
Late rice	19.64	Tobacco	26
Single cropping rice	17.24	Tea	24
Barley	24.07	Bast fibre plants	62
Foxtail millet	20.07	Cotton	60.03
Sorghum	28.97	Sugar beet	7.63
Other cereals	26.8	Sugarcane	3.4
Potato	10.6	Soybeans	39.05
Sweet potato	7.13	Pea	28.6

#### Leaching and runoff outputs

Few reports have been published quantifying accurate amounts of K_2_O leaching and runoff outputs. This type of output only accounted for a small percentage of the total outputs. In this research, the values for this parameter were available in S3 Table [32].

## Results

### Temporal and spatial variation in K inputs

#### Temporal variation in K input in China

**Fig 1:**
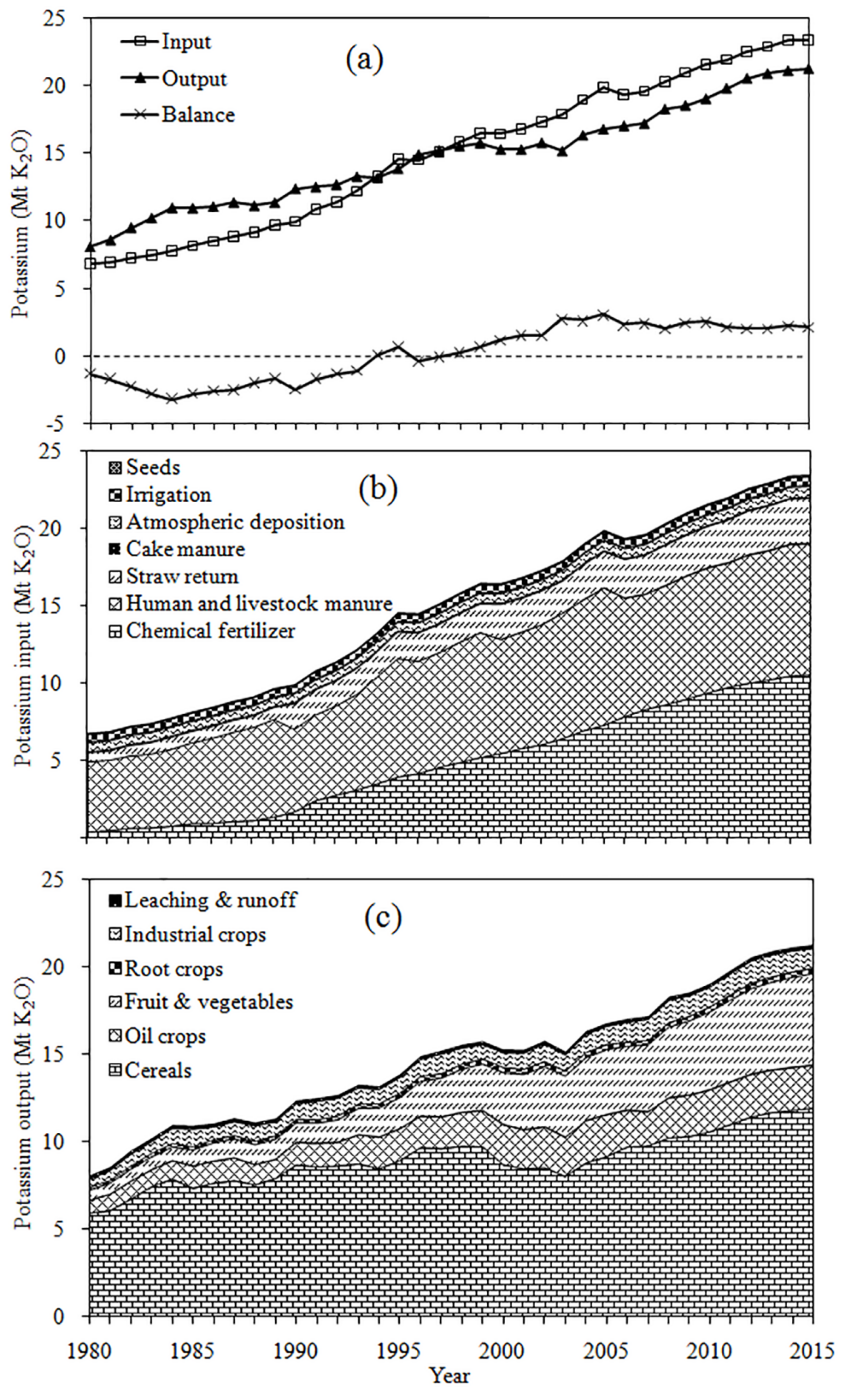
The total amounts of potassium input, output and balance (a), and temporal variation of compositions of K input (b) and output (c) in agricultural land for China from 1980 to 2015.

At the national scale, the average K input from 1980 to 2015 was 14.95 Mt K_2_O (146.92 kg K_2_O ha^-1^ yr^-1^). The K input showed an increasing trend with an average annual increment of 0.48 Mt K_2_O (3.94 kg K_2_O ha^-1^ yr^-1^) from 6.78 Mt K_2_O in 1980 to 23.44 Mt K_2_O in 2015 with the coefficient of determination (R^2^) of 0.990 (Fig 1A). At the national level, the K inputs all increased considerably with time; the K inputs were 8.03 Mt K_2_O (84.25 kg K_2_O ha^-1^ yr^-1^), 13.41 Mt K_2_O (139.35 kg K_2_O ha^-1^ yr^-1^), 18.78 Mt K_2_O (183.32 kg K_2_O ha^-1^ yr^-1^) and 22.66 Mt K_2_O (203.33 kg K_2_O ha^-1^ yr^-1^) in the 1980s, 1990s, 2000s and 2010s, respectively.

**Fig 2:**
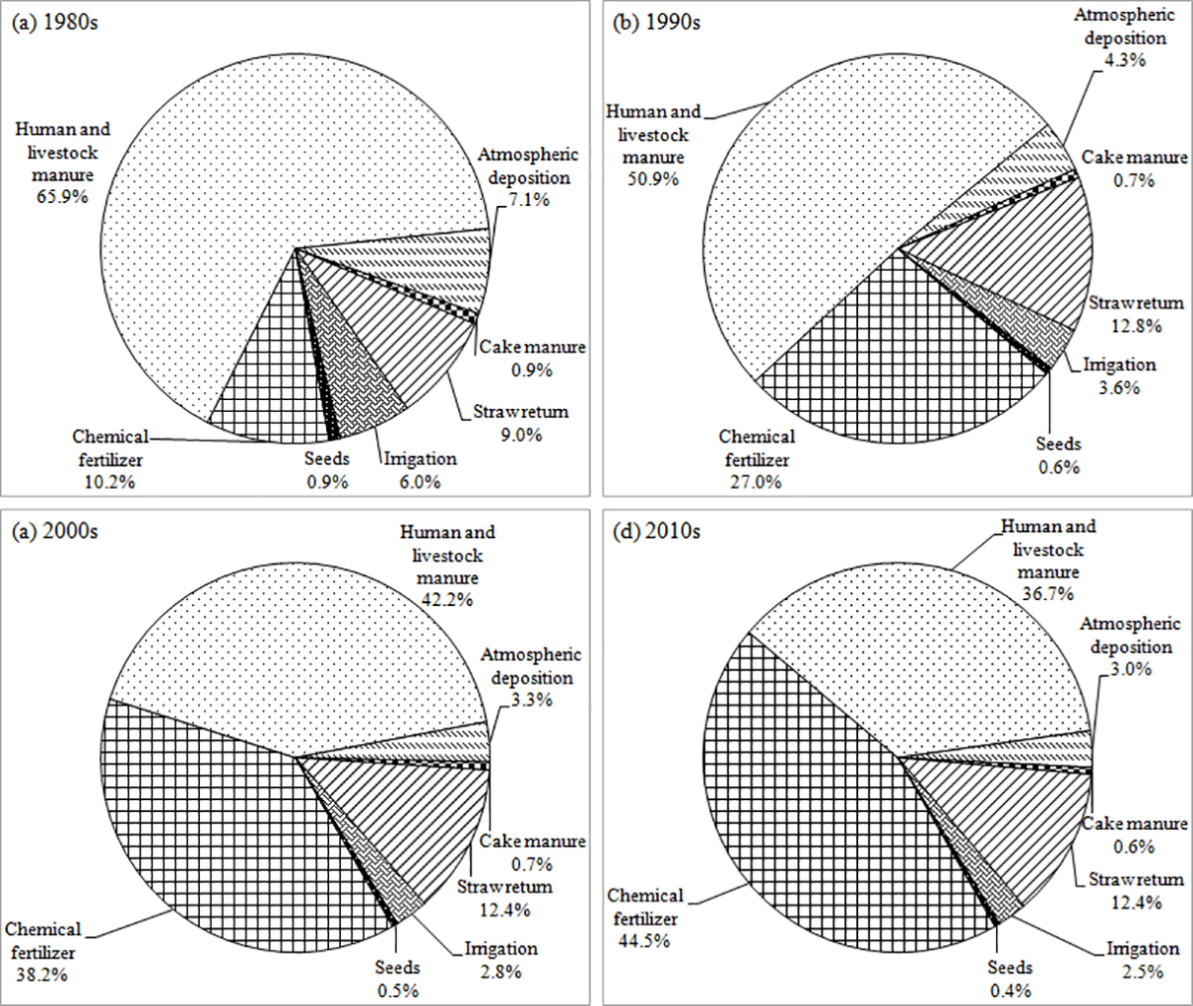
Composition of the total K_2_O input in agricultural land for China in 1980s, 1990s, 2000s and 2010s.

The K input mainly consisted of three components: chemical fertilizer, organic manure and other K input resources (atmospheric deposition, irrigation and seeds), which accounted for 32.8% (4.91 Mt K_2_O), 59.2% (8.85 Mt K_2_O) and 8.0% (1.19 Mt K_2_O) of the total input from 1980 to 2015, respectively. The K input of chemical fertilizer increased dramatically over time with an average annual increment of 0.29 Mt K_2_O from 0.38 Mt K_2_O in 1980 to 10.58 Mt K_2_O in 2015. Additionally, the K input of human and livestock manure and straw return increased substantially from 1980 to 2015 with average annual increments of 0.11 and 0.07 Mt K_2_O, respectively (Fig 1B). In the past four decades, the K input from chemical fertilizer became a larger contributor accounting for 10.2, 27.0, 38.2 and 44.5% of total K input, respectively. The K input from human and livestock manure accounted for a substantial proportion of the total K input, but it showed a decreasing trend which was 65.9, 50.9, 42.2 and 36.7% in the 1980s, 1990s, 2000s and 2010s, respectively. At the same time, the ratio of straw return increased over time, which was 9.0, 12.8, 12.4 and 12.4% in the 1980s, 1990s, 2000s and 2010s, respectively. However, the K input from irrigation, atmospheric deposition, cake manure and seeds was small and decreased over time (Fig 2).

#### Temporal and spatial variation in K input in six regions

**Fig 3:**
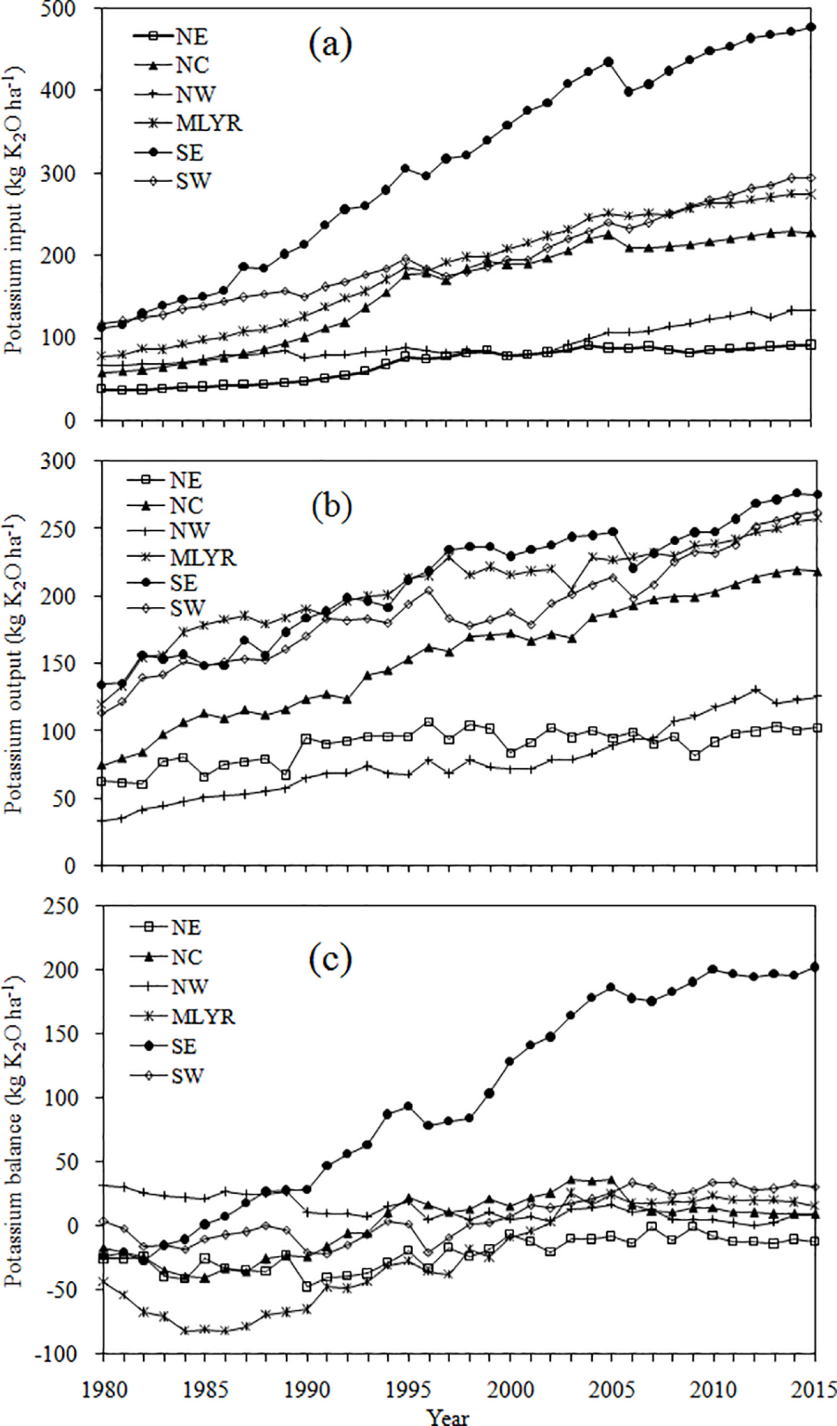
Temporal variation of K input (a), output (b) and balance (c) in the unit of cultivated land in six regions from 1980 to 2015.

The K input in six regions all increased over time, despite two fluctuations in 1995 and 2005 (Fig 3A). At the regional level, the K input in SE was the highest and increased rapidly from 111.65 kg K_2_O ha^-1^ in 1980 to 477.56 kg K_2_O ha^-1^ in 2015, followed by the NC, MLYR and SW which increased from 58.24, 77.01 and 117.10 kg K_2_O ha^-1^ in 1980 to 228.15, 273.67 and 293.86 kg K_2_O ha^-1^ in 2015, respectively. The K input in the NE and NW increased slowly and steadily from 37.34 and 65.67 kg K_2_O ha^-1^ in 1980 to 90.91 and 134.06 kg K_2_O ha^-1^ in 2015, respectively.

**Fig 4:**
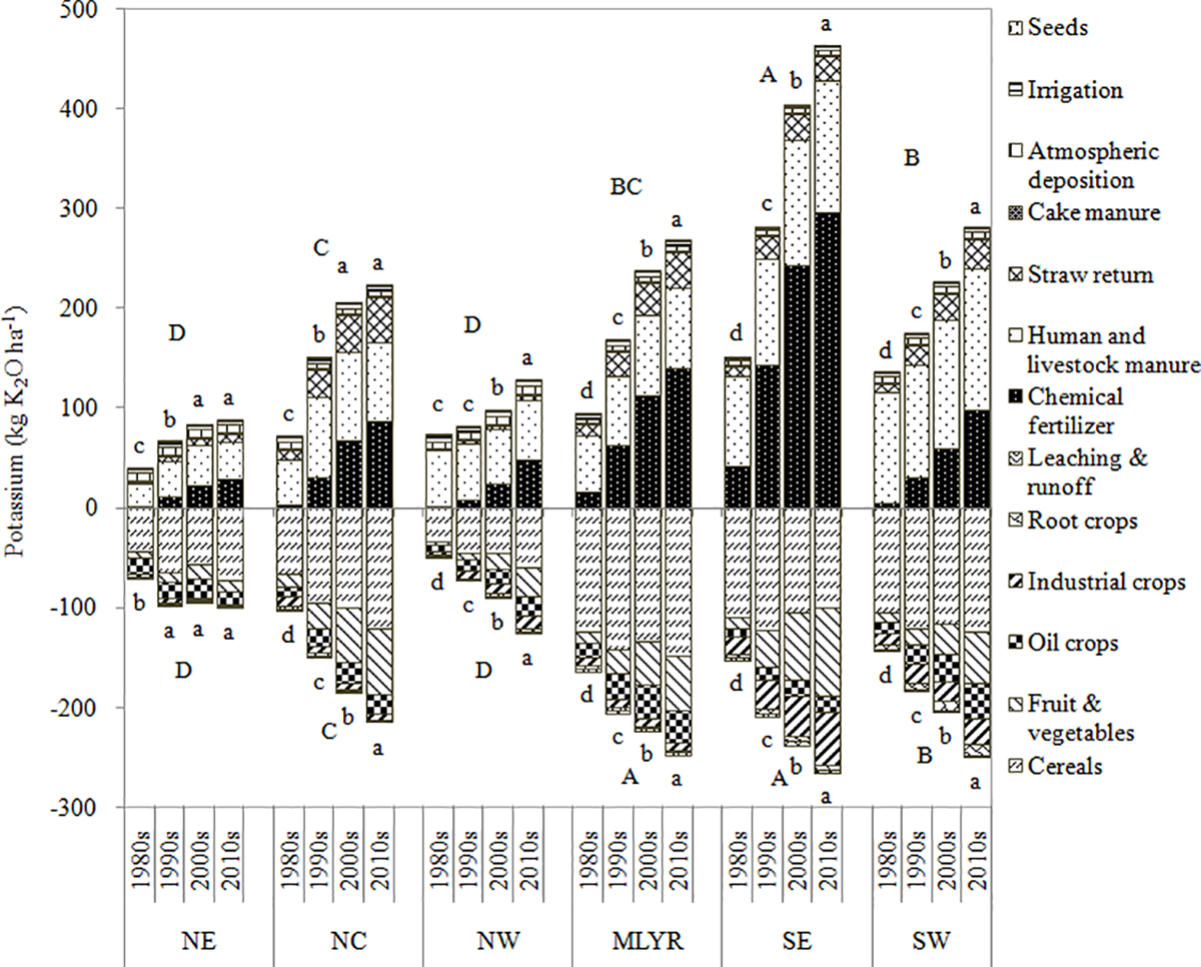
Potassium inputs and outputs in agricultural land in six regions in 1980s, 1990s, 2000s and 2010s (positive values represented K inputs and negative values meant the K outputs).

The significant variation in K input at the regional level can be easily observed (positive values in Fig 4). The K input in SE was significantly larger than that in other regions. The K input in SW was significantly larger than that in the NE, NC and NW, but it was not significantly different from that in MLYR. Besides, the K input in the MLYR was significantly larger than those in NE and NW, but it was not significantly different from that in the NC. In addition, The K input in the NC was significantly larger than those in NE and NW. There was no significant difference in K input between NE and NW. The K input in all six regions increased significantly over the past four decades, except for the K input in the NE and NC that was maintained the same level in the 2000s and 2010s, and the K input in the NW showed no increase between 1980s and 1990s. The SE had the largest K inputs which were 152.05, 282.60, 405.38 and 464.00 kg K_2_O ha^-1^ in 1980s, 1990s, 2000s and 2010s, respectively. Additionally, the NE and NW had the lowest inputs which were 40.25, 67.09, 84.60, 88.34 and 73.72, 82.30, 98.36, 128.87 kg K_2_O ha^-1^ in 1980s, 1990s, 2000s and 2010s, respectively (Fig 4).

At the regional level, all input flow types with positive values in six regions over the past four decades were displayed in Fig 4. The K input from chemical fertilizer was more than that from manure in NE, NC, NW and SW except that in NC in the 2010s, in which the K input from chemical fertilizer (2.37 Mt K_2_O, 87.55 kg K_2_O ha^-1^ yr^-1^) was more than that from human and livestock manure (2.11 Mt K_2_O, 78.21 kg K_2_O ha^-1^ yr^-1^). The K input in the 1980s and 1990s of the MLYR was mainly derived from human and livestock manure, with values of 1.16 Mt K_2_O (56.87 kg K_2_O ha^-1^ yr^-1^) and 1.37 Mt K_2_O (68.45 kg K_2_O ha^-1^ yr^-1^), respectively. However, the K input in the 2000s and 2010s of the MLYR was primarily chemical fertilizer, with values of 2.28 Mt K_2_O (113.10 kg K_2_O ha^-1^ yr^-1^) and 2.97 Mt K_2_O (139.56 kg K_2_O ha^-1^ yr^-1^), respectively. Most of the K input in the SE was from chemical fertilizer except in the 1980s when the K input was chiefly from human and livestock manure (0.64 Mt K_2_O, 90.96 kg K_2_O ha^-1^ yr^-1^).

### Temporal and spatial variation in K output

#### Temporal and spatial variation in K output in China

At the national scale, the average K output from 1980 to 2015 was 14.78 Mt K_2_O (146.11 kg K_2_O ha^-1^ yr^-1^). Additionally, the K output increased substantially over time with an average annual increment of 0.38 Mt K_2_O (3.01 kg K_2_O ha^-1^ yr^-1^) from 8.10 Mt K_2_O in 1980 to 21.31 Mt K_2_O in 2015 with a determination coefficient (R^2^) of 0.976 (Fig 1A). At the national level, the K output increased considerably over time with values of 108.41, 145.27, 162.01 and 183.84 kg K_2_O ha^-1^ in the 1980s, 1990s, 2000s and 2010s, respectively.

**Fig 5:**
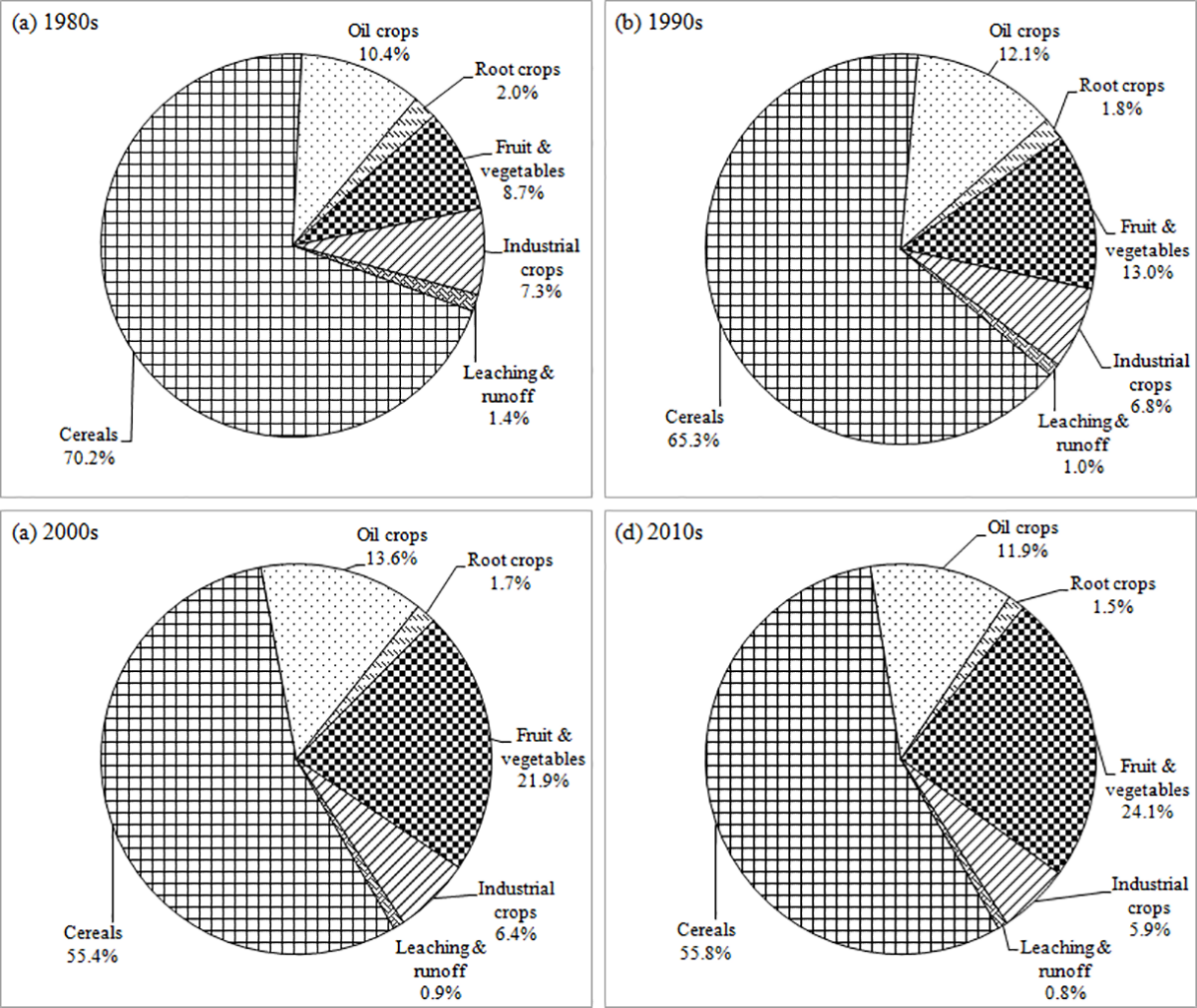
Composition of the total K_2_O output in agricultural land for China in 1980s, 1990s, 2000s and 2010s.

The K output mainly consisted of two components: crops removal and loss (leaching and runoff), which accounted for 99.0% (14.63 Mt K_2_O) and 1.0% (0.15 Mt K_2_O) of total output, respectively from 1980 to 2015. The K output of cereals crops increased dramatically over time with an average annual increment of 0.17 Mt K_2_O from 5.95 Mt K_2_O in 1980 to 11.95 Mt K_2_O in 2015. Additionally, the K output of fruit and vegetables and oil crops increased substantially from 1980 to 2015 with average annual increments of 0.13 and 0.05 Mt K_2_O, respectively (Fig 1C). The K output from crops removal of cereals was the most important part of the output, accounting for 70.2, 65.3, 55.4 and 55.8% in 1980s, 1990s, 2000s and 2010s, respectively. The K output from crops removal of fruit and vegetables gradually became an important part of the output accounting for 8.7, 13.0, 21.9 and 24.1% in the 1980s, 1990s, 2000s and 2010s, respectively. In addition, the crops removal of oil crops (which accounted for 10.4, 12.1, 13.6 and 11.9% of the K output) and industrial crops (which accounted for 7.3, 6.8, 6.4 and 5.9% of the K output) were relatively steady in the 1980s, 1990s, 2000s and 2010s, respectively. The crops removal of root crops and leaching & runoff accounted for a small part of the total K output and decreased over time (Fig 5).

#### Temporal and spatial variation in K output by regions

Although the output had some fluctuations, the trend in K output generally increased over time in all regions (Fig 3B). At the regional level, the K output in the MLYR, SE and SW was high in 2015 and fluctuated suddenly (increasing from 120.25, 135.02 and 113.35 kg K_2_O ha^-1^ in 1980 to 257.74, 275.28 and 262.60 kg K_2_O ha^-1^ in 2015), followed by the NC (increasing from 74.77 kg K_2_O ha^-1^ in 1980 to 218.70 kg K_2_O ha^-1^ in 2015), and then the NE and NW (increasing from 62.78 and 33.65 kg K_2_O ha^-1^ in 1980 to 102.80 and 125.85 kg K_2_O ha^-1^ in 2015). Nevertheless, the K output in the NE increased slowly with some fluctuations from 1982 to 1989, thereafter stabilizing from 1990 (94.47 kg K_2_O ha^-1^) to 2015.

There were significant variations in K output at the regional level (negative values in the Fig 4). The K outputs in MLYR and SE were significantly larger than those in other regions. The K output in SW was significantly larger than that in NE, NC and NW. In addition, the K output in NC was significantly larger than that in NE and NW. There was no significant variation in K output between NE and NW. The K outputs all increased significantly over the past four decades except for NE which increased from the 1980s to the 1990s, but it didn’t change from the 1990s to the 2010s. The MLYR and SE had large K outputs which were 164.86, 207.03, 224.70, 248.69 and 153.57, 210.03, 237.98, 266.09 kg K_2_O ha^-1^ in the 1980s, 1990s, 2000s and 2010s, respectively. Additionally, the NE and NW had the lowest K outputs with values of 70.97, 97.27, 93.85, 99.63 and 47.70, 71.71, 88.56, 124.03 kg K_2_O ha^-1^ in the 1980s, 1990s, 2000s and 2010s, respectively (Fig 4).

The composition of K output at the regional level differed among regions, which was revealed by the negative values (Fig 4). The main K output source was cereals crops, which increased over time for all regions except for SE. As for the other output sources, the K output from fruit & vegetables and oil crops accounted for a larger part for all regions except for SE in which the larger sources of K the output were fruit & vegetables and industrial crops.

### Temporal and spatial variation in K balance

#### Temporal variation in K balance in China

At the national scale, the average K balance from 1980 to 2015 was 0.81 kg K_2_O ha^-1^ yr^-1^. K was deficient from 1980 (-1.33 Mt K_2_O) to 1997 (-0.10 Mt K_2_O), but this condition was gradually alleviated from 1984 (-3.23 Mt K_2_O) to 1995 (0.67 Mt K_2_O). After that time, the K balance reached a slight surplus and remained relatively stable from 2003 (2.74 Mt K_2_O) to 2015 (2.13 Mt K_2_O) (Fig 1A). On average, K balance increased by -24.17, -5.92, 21.31 and 19.50 kg K_2_O ha^-1^ in the 1980s, 1990s, 2000s and 2010s, respectively.

#### Temporal and spatial variation in K balance by regions

As it was shown in the lie graph (Fig 3C), the average K balance in six regions from 1980 to 2015 varied widely with values of -21.37, 1.25, 13.70, -22.79, 99.22 and 7.18 kg K_2_O ha^-1^, respectively. The annual K balance showed different trends in six regions (Fig 3C). The K balance in the SE moved into surplus from 1985 onwards, while the K balance in NE remained in the negative changing from -25.44 kg K_2_O ha^-1^ in 1980 to -11.90 kg K_2_O ha^-1^ in 2015. Additionally, other four regions all reached balance by 2002. In detail, the balance in the SE increased from 1980 to 2005 (186.88 kg K_2_O ha^-1^) even though the K balance had a short term decrease from 1995 to 1998; after 2005 it remained relatively stable to 2015 (202.29 kg K_2_O ha^-1^). In addition, the balance in the NW was slightly in surplus and remained relatively stable from 1980 (32.02 kg K_2_O ha^-1^) to 2015 (8.21 kg K_2_O ha^-1^). The balance in the NC and SW was negative from 1980 (-16.53 and 3.74 kg K_2_O ha^-1^, respectively) to 1993 (-5.33 and -6.66 kg K_2_O ha^-1^, respectively) and in the MLYR from 1980 (-43.23 kg K_2_O ha^-1^) to 2001 (-3.62 kg K_2_O ha^-1^), after which they were all in surplus until 2015 (9.45, 31.26 and 15.94 kg K_2_O ha^-1^ in NC, SW and MLYR, respectively). To summarize, the negative stage of K balance could be divided into two stages: firstly a decrease and then an increase.

**Fig 6:**
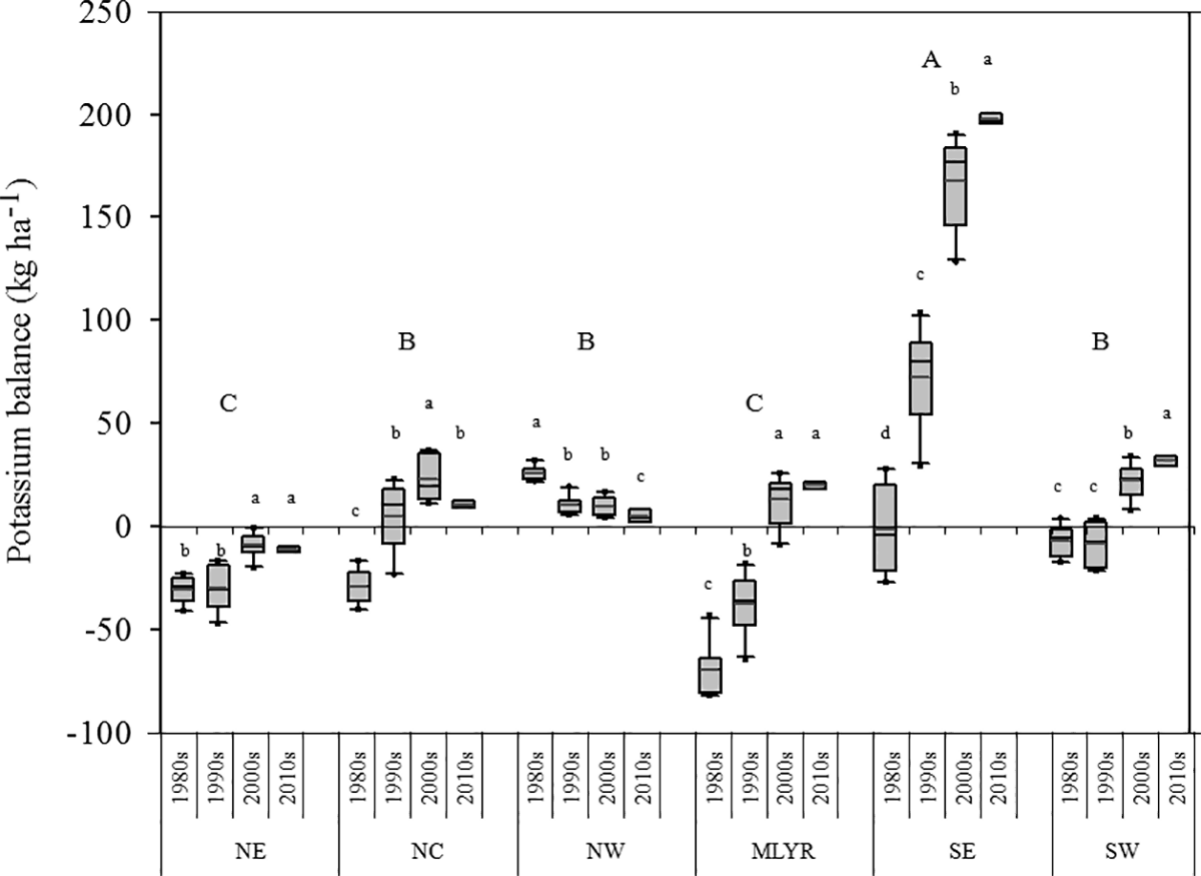
Potassium balance in agricultural land in six regions in 1980s, 1990s, 2000s and 2010s.

The average K balance in SE was the largest and had a distinct increase over time with values of -1.53, 72.58, 167.40 and 197.91 kg K_2_O ha^-1^ in the 1980s, 1990s, 2000s and 2010s, respectively. However, the K balances in NE and MLYR were significantly lower than those in other regions with values of -30.72, -30.18, -9.25, -11.29 and -69.41, -37.74, 13.14, 19.91 kg K_2_O ha^-1^ in the 1980s, 1990s, 2000s and 2010s, respectively; NE stayed in deficit for the entire period. The K balances in NE, MLYR, SE and SW all increased significantly with time. Although NW remained in surplus throughout the study period the values decreased over time with values of 26.02, 10.60, 9.80 and 4.84 kg K_2_O ha^-1^in the 1980s, 1990s 2000s and 2010s, respectively. At the meantime, the K balance in NC increased from the 1980s (-29.08 kg K_2_O ha^-1^) to the 2000s (22.64 kg K_2_O ha^-1^), and then decreased dramatically between the 2000s and the 2010s (10.72 kg K_2_O ha^-1^) (Fig 6).

#### Temporal and spatial variation of KUE

**Fig 7:**
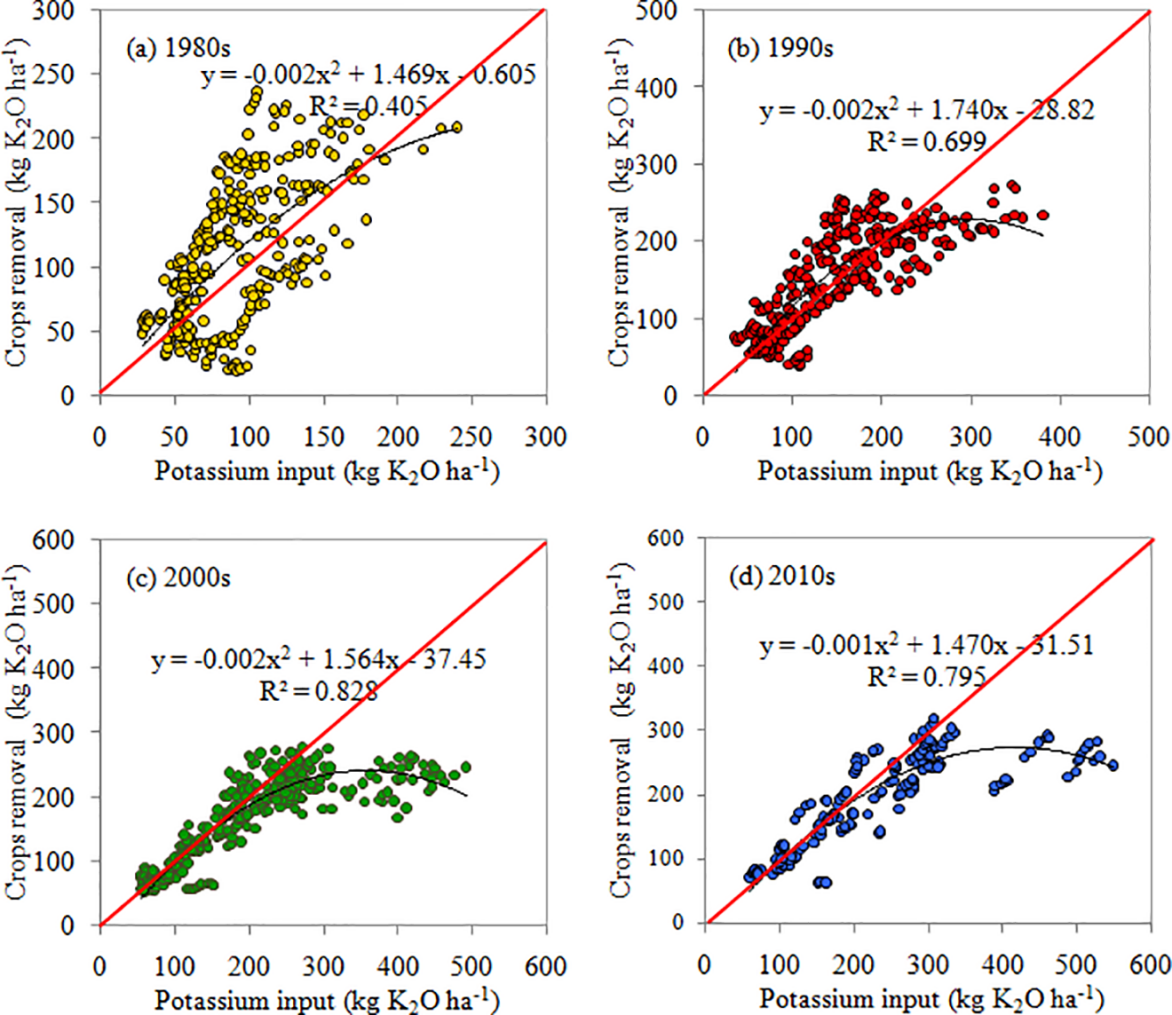
Potassium use efficiency (KUE), calculated as the crops removal (K output in crops) divided by the total K input of a system (a) in 1980s, (b) in 1990s, (c) in 2000s and (d) in 2010s. Each point respected a province. The red line meant 1:1 line.

Average KUE in China from 1980 to 2015 was 103.60%. During the past four decades, the KUE decreased with values of 127.09, 104.35, 87.69 and 89.69% (Table 4), and the decline in the slope could also reflect the KUE (Fig 7). However, great variation in KUE existed across different regions with mean values of 138.63, 107.28, 83.02, 121.65, 73.97 and 97.22% for NE, NC, NW, MLYR, SE and SW, respectively (Table 3).

**Table 4:** The potassium use efficiency (KUE) in six regions during the past four decades.

Items	NE	NC	NW	MLYR	SE	SW	CHINA
	------%------						
1980s	174.02	140.84	64.19	168.67	100.88	103.72	127.09
1990s	148.26	99.20	87.03	121.57	73.57	103.83	104.35
2000s	109.80	89.06	89.96	93.06	57.93	89.62	87.69
2010s	111.63	95.16	96.19	91.08	56.53	88.01	89.69
Average	138.63	107.28	83.02	121.65	73.97	97.22	103.60

There was a positive linear correlation between K input and K output in crops with the correlation coefficients (R^2^) of 0.41, 0.70, 0.83 and 0.80 for the 1980s, 1990s, 2000s and 2010s, respectively (Fig 7). The balanced condition could also be found according to the 1:1 line, which was divided into two parts. Left part of the 1:1 line represented K deficit, which meant that the K output was larger than the K input. On the contrary, right part of the 1:1 line represented K surplus which meant that the K input was larger than the K output. If the points were around the line, it meant that the K in some places had reached to a balance stage. Additionally, data points in the 1980s were scattered largely above and below the 1:1 line while more data points in the 1990s aggregated around the line; more data points were below the line in the 2000s and 2010s.

## Discussion

### Temporal and spatial variability in the K balance

There was great spatial variation for K input and output which was correlated with the different crops and soil fertility [30, 43]. Results in this study indicated that K input and output increased dramatically with time. The K input kept increasing as the use of chemical fertilizer increased year by year, whereas the K output also continued to increase because of the increase in crop yield and change of K output from different crops. For example, the proportion of K removal in output for cereals crops (low K uptake) decreased from 70.2% to 55.8%, but it increased from 8.7% to 24.1% for fruit & vegetables (high K uptake) in the past four decades. However, the input and output in the NW were lower over the past four decades than those in other regions because of the low chemical K fertilizer application and low crop yields [43, 44]. On one hand, over-reliance on the imported K fertilizer and the increasing cost limited the use of K fertilizer [13, 24, 45]. On the other hand, some farmers believed that N and P application played a more important role in increasing crop yield than K fertilizer [46] because the application of K fertilizer does not produce results as quickly as N and P fertilizer application; this is because of the high soil K supply capacity with high K-containing minerals such as micas and feldspars in the soil parent material [47, 48].

Conversely, the K balance increased steadily, which was mainly attributed to improvement of the social economy and improved management among farmer households [3]. For example, chemical fertilizer application in the SE increased dramatically with economic development, leading to a substantial surplus in the K balance. Another important reason for the improved K balance is that more crop residues were returned back to soils with the development of agricultural mechanization [43]. In addition, K balance in the NE was still deficient which indicated that K input in the NE was insufficient to meet crop production. However, the soil available K in NE was stable from the 1990s to the 2000s [43], which implied that the above-mentioned K input sources were not the only source of K for crop absorption and that soil K mineralization also played a substantial role. In this study, the K balance in the NW was stable at the equilibrium state over four decades, but the soil available K decreased from the 1990s to the 2000s [43]. One of the reasons for this might because K was easily fixed with the alternation of dry and wet climate conditions [49]; this might result in decreasing K availability because of the slow mineralization of returned straw [50, 51].

### K fertilization predictions and recommendations

The nutrient balance exhibited a strong relationship with nutrient management and sustainable agricultural development [3], so it was necessary to budget the agricultural nutrient balance on a macro level, especially for policy makers who need to be informed on national situations. The K balance prediction in 2020 was mainly based on the policy of zero growth of chemical fertilizer and 60% straw and manure return rates. So far, the crop yield in 2015 was the highest and could be regarded as the optimal level. We assumed that if zero growth of chemical fertilizer is achieved by 2020 and crop yields is maintained at the optimal level as those in 2015, the K balance for China will be 76.50 kg K_2_O ha^-1^. Large temporal and spatial variation in the K balance highlighted that fertilizer application and management practices need to be adjusted based on local conditions. For example, the K surplus in the SE was over 70% in recent years, which demonstrated that the potential for decreasing K fertilizer application in SE was large. Therefore, it is realistic to decrease chemical K fertilization whilst maintaining high and steady yields without increasing cropping areas. Furthermore, reducing the rate of K fertilizer applied was effective in reducing K surplus and enhancing the KUE [52]. The K fertilizer application could be decreased by 202.29 kg K_2_O ha^-1^ to maintain equilibrium in the K balance. In contrast, it is essential to apply more K fertilizer timely and efficiently in both NE and NW in order to maintain the soil K balance over the long-term because of the decline in the K supply capacity. Reports indicated that straw return alone was not sufficient to maintain the soil K balance although more crop residues were returned back to soils with the development of agricultural mechanization [47, 53, 54]. Nevertheless, it is necessary to pay attention to the rational utilization of organic manure, especially cow urine in the field, and combine it with the same rate of commercial fertilizer in order to alleviate the K deficit [3, 18]. The K balance in the SE showed a short-term decrease from 1995 to 1998 because of the reduction in the number of cattle, which indicated that cow urine contributed substantially to the K balance. Certainly, nutrient management recommendations must change with yield levels and profit maximization in crop production systems [55].

### KUE

Potassium use efficiency (KUE) is the uptake efficiency defined as the percentage of K input that is recovered as K output in crops [3, 12, 34]. The KUE in China decreased with time because of increasing K application rate which suggested that the effect of K fertilizer on increasing grain production had diminished [55, 56]. From the distribution of data in Fig 7, the soil K balance in the 1990s in China was the most reasonable period during the past four decades, which was in accordance with the K balance results (-24.17, -5.92, 21.31 and 19.50 K_2_O ha^-1^ in the 1980s, 1990s, 2000s and 2010s, respectively). Nevertheless, the K balances went into surplus in several provinces in the 2000s and 2010s, which indicated that K fertilization has deserved more attention with time. However, great spatial variability in KUE existed with values of 138.63, 107.28, 83.02, 121.65, 73.97 and 97.22% in the 2010s for NE, NC, NW, MLYR, SE and SW, respectively. The KUE in SE was the lowest because of the overuse of K fertilizer.

## Conclusions

It is necessary to budget the soil K nutrient balance on the national level, especially for policy makers. The tendency toward N and P surplus and K deficit has been addressed and corrected, but large spatial variation in the K balance in this study highlighted that fertilizer application and management practices need to be adjusted depending on local conditions. Well-managed fertilizer use can create a “win-win” situation by increasing food production and reducing soil degradation in nutrient-poor, fragile soils. Increased K fertilizer application has led to a reduction in fertilizer use efficiency in China. There is an urgent need for the government to act decisively on this issue. The effect of K balance on crop yields under different soil fertility should be verified in different soil types in further studies, to continue the application and extension of these results. Therefore, K fertilizer management needs to consider the soil K balance so as to build up a soil K pool to guarantee both high yield and high efficiency of K fertilizer. It is hoped that these results will provide a basis for rational K nutrient management in the Chinese agricultural system.

## Supporting information

S1 Table The proportion K_2_O fertilizer in compound fertilizer at six regions in China. (PDF)

S2 Table The cake production rate of different crops and the K content in the cake manure. (PDF)

S3 Table Other input resources (deposition, irrigation and seeds) and loss rate (leaching and runoff). (PDF)
