# Supplementary material for: Temporal and spatial variation of potassium balance in agricultural land at national and regional levels in China
Source: PLoS One. 2017 Sep 5;12(9):e0184156. doi: 10.1371/journal.pone.0184156 (PMC5584956; doi:10.1371/journal.pone.0184156)
Supplement: S1 Table — (PDF) [file pone.0184156.s001.pdf]

S1 Table The proportion K_2O fertilizer in compound fertilizer at six regions in China.

Regions	NE	NC	NW	MLYR	SE	SW
Provinces	HLJ, JL, LN	BJ, HEB, HEN, SD, SX, TJ	GS, NMG, NX, QH, SNX,XJ	AH, HUB, HUN, JS, JX, SH, ZJ	FJ, GD, GX, HN	CQ, GZ, SC, XZ, YN,
1980	1:3.60:0.12	1:2.70:0.25		1:1.80:0.49		
1981	1:3.54:0.13	1:2.66:0.25		1:1.77:0.50		
1982	1:3.94:0.09	1:2.96:0.19		1:1.97:0.37		
1983	1:4.04:0.09	1:3.03:0.17		1:2.02:0.34		
1984	1:4.26:0.07	1:3.20:0.14		1:2.13:0.27		
1985	1:4.04:0.09	1:3.03:0.18		1:2.02:0.35		
1986	1:3.94:0.08	1:2.96:0.16		1:1.97:0.31		
1987	1:3.90:0.08	1:2.93:0.16		1:1.95:0.32		
1988	1:4.28:0.06	1:3.21:0.12		1:2.14:0.24		
1989	1:4.14:0.07	1:3.11:0.14		1:2.07:0.27		
1990	1:4.02:0.08	1:3.02:0.16		1:2.01:0.31		
1991	1:2.18:0.17	1:1.64:0.35		1:1.09:0.69		
1992	1:2.16:0.17	1:1.62:0.35		1:1.08:0.69		
1993	1:1.98:0.18	1:1.49:0.36		1:0.99:0.72		
1994	1:1.92:0.19	1:1.44:0.37		1:0.96:0.74		
1995	1:1.98:0.18	1:1.49:0.36		1:0.99:0.72		
1996	1:2.18:0.17	1:1.64:0.35		1:1.09:0.69		
1997	1:2.26:0.17	1:1.70:0.34		1:1.13:0.67		
1998	1:2.16:0.17	1:1.62:0.35		1:1.08:0.69		
1999	1:2.14:0.18	1:1.61:0.35		1:1.07:0.70		
2000	1:2.02:0.18	1:1.52:0.36		1:1.01:0.72		
2001	1:2.06:0.18	1:1.55:0.36		1:1.03:0.71		
2002	1:2.14:0.17	1:1.61:0.35		1:1.07:0.69		
2003	1:2.00:0.18	1:1.50:0.36		1:1:0.72		
2004	1:2.26:0.20	1:1.70:0.40		1:1.13:0.79		
2005	1:2.20:0.19	1:1.65:0.38		1:1.10:0.76		
2006-2015	1:2:0.2	1:1.5:0.4		1:1:0.80		

[a] From: Yan (2008); Li and Jin (2011)

[b] Six regions were grouped based on geographical locations and China's administrative divisions (Li and Jin, 2011). NE, northeast; NC, north central; NW, northwest; MLYR, the middle and lower reaches of the Yangtze River; SE, southeast; SW, southwest. AH, Anhui; BJ, Beijing; CQ, Chongqing; FJ, Fujian; GD, Guangdong; GS, Gansu;

GX, Guangxi; GZ, Guizhou; HEB, Hebei; HEN, Henan; HLJ, Heilongjiang; HN, Hainan; HUB, Hubei; HUN, Hunan; JL, Jilin; JS, Jiangsu; JX, Jiangxi; LN, Liaoning; IM, Inner Mongolia; NX, Ningxia; QH, Qinghai; SC, Sichuan; SD, Shandong; SH, Shanghai; SNX, Shaanxi; SX, Shanxi; TB, Tibet; TJ, Tianjin; XJ, Xinjiang; YN, Yunnan; ZJ, Zhejiang. The above provinces don’t include Hong Kong, Macao and Taiwan. CQ was started from 1997.
