# Supplementary material for: Temporal and spatial variation of potassium balance in agricultural land at national and regional levels in China
Source: PLoS One. 2017 Sep 5;12(9):e0184156. doi: 10.1371/journal.pone.0184156 (PMC5584956; doi:10.1371/journal.pone.0184156)
Supplement: S2 Table — (PDF) [file pone.0184156.s002.pdf]

S2 Table The cake production rate of different crops and the K content in the cake manure.

Crops	Cake rate [a]	Content [b] (%)
Beans	0.85	1.434
Peanut	0.5	1.157
Rapeseed	0.55	1.253
Sunflower seed	0.7	1.591
Cotton seeds	0.8	0.916
Sesame seeds	0.5	0.675
Flax	0.7	1.326

[a] From: Zhang et al. (2010b)

[b] From: China Agric. Press (1999a)
