# Supplementary material for: Temporal and spatial variation of potassium balance in agricultural land at national and regional levels in China
Source: PLoS One. 2017 Sep 5;12(9):e0184156. doi: 10.1371/journal.pone.0184156 (PMC5584956; doi:10.1371/journal.pone.0184156)
Supplement: S3 Table — (PDF) [file pone.0184156.s003.pdf]

S3 Table Other input resources (deposition, irrigation and seeds) and loss rate (leaching and runoff).

Item	Unit	NE	NC	NW	MLYR	SE	SW
Deposition [1]	(kg K_2O ha^{-1})	8.2	5.3	6.7	4.5	4.4	6.8
Irrigation [1]	(kg K_2O ha^{-1})	4.3	5.8	6	5.3	4	3.2
Seeds [1]	(kg K_2O ha^{-1})	0.79	0.6	1.0	0.43	0.2	0.68
Leaching & runoff [1]	(kg K_2O ha^{-1})	1.0	0.1	0.1	4.0	3.7	2.0

[a] Li and Jin (2011)
